# Supplementary material for: Predictors of puerperal menstruation
Source: PLoS One. 2020 Jul 10;15(7):e0235888. doi: 10.1371/journal.pone.0235888 (PMC7351153; doi:10.1371/journal.pone.0235888)
Supplement: Attachment — Submitted filename: RETURN OF MENSES-2ND REPLY TO [JUNE 2020] REVIEWERS-PONE.docx [file pone.0235888.s003.docx]

23-06-2020

From

Corresponding author (George Eleje)

To

Editor

PLOS ONE

Dear editors:

Re: Submission of Response to Editors and Reviewers’ comments on Manuscript ID (Submission ID: PONE-D-19-23514R1) entitled "Predictors of puerperal menstruation”.

Please find enclosed a point-by-point response to the comments by the editors. We hope that the editors and reviewers will find the revisions acceptable.

Dear Dr. ELEJE,

Thank you for submitting your manuscript to PLOS ONE. After careful consideration, we feel that it has merit but does not fully meet PLOS ONE’s publication criteria as it currently stands. Therefore, we invite you to submit a revised version of the manuscript that addresses the points raised during the review process.

==============================

Both reviewers agree that the revised version of the manuscript has satisfied previous doubts and that it meets PLOS ONE's publication criteria. After my evaluation of the manuscript, the following changes are required for acceptance.

- Abstract:  “childbirth” instead of “child birth”

**AUTHORS’ RESPONSE**

As suggested, we have now effected the changes needed.

- Last sentence of introduction: “This study therefore IS AIMED AT DETERMINING..”

**AUTHORS’ RESPONSE**

As suggested, we have now effected the changes needed.

- M&M, line 10: close parentheses:  (time at resumption of menses and coitus postpartum) .

**AUTHORS’ RESPONSE**

As suggested, we have now effected the changes needed.

- M&M Line 19: “validation. Firstly” instead of “validation: Firstly”. “Face validity of the questionnaire”

**AUTHORS’ RESPONSE**

As suggested, we have now effected the changes needed.

- page 10, line 24; remove  “given a ratio of 1:3”

**AUTHORS’ RESPONSE**

As suggested, we have now effected the changes needed.

- page 14, line 21, and following: “breastfeeding” instead of “breast feeding”

**AUTHORS’ RESPONSE**

As suggested, we have now effected the changes needed.

- page 15, line 3-4. Check spaces and –suboptimal

**AUTHORS’ RESPONSE**

As suggested, we have now effected the changes needed.

- page 15,line 20:   “counterpart” instead of “counter-part”

**AUTHORS’ RESPONSE**

As suggested, we have now effected the changes needed.

- discussion: “The main strength of this study was that this study appears to be the first study that attempts to describe puerperal menstruation. However, we could not by this study validate the determinants of other variables such as nutritional status of the women which could increase the risk of early return of menses [4, 5].”. I suggest to remove these sentences  and to begin the paragraph with: “The main limitation of our study is... “
- conclusion: remove “this study appears to be the first study to date that attempts..” and begin the paragraph with “Our data show that..”

**AUTHORS’ RESPONSE**

As suggested, we have now effected the changes needed. For instance: We have concluded as follows: In conclusion, our data show that approximately one third of the parturients achieve puerperal menstruation and the significant associated risk factors include younger age, multiparity, early use of family planning, high socio-economic status, ‘exclusive breastfeeding’ and manual removal of placenta. Of these, the major predictor was early initiation of family planning, while exclusive breastfeeding with manual removal of placenta a major protective factor. These study findings could be useful to understand among the clinicians and patients the importance to increase prevention strategies aimed at avoiding unintended pregnancies during the postpartum period, especially in younger women. This study has raised in­teresting issues and requires further investigation to better understand the mechanism of puerperal menstruation.

- in table 3, indicate the ref value as suggested by Reviewer #1 (see below)

**AUTHORS’ RESPONSE**

As suggested, we have now indicated the reference value (Reference) as needed in table 3.

==============================

Please submit your revised manuscript by Aug 01 2020 11:59PM. If you will need more time than this to complete your revisions, please reply to this message or contact the journal office at plosone@plos.org. When you're ready to submit your revision, log on to https://www.editorialmanager.com/pone/ and select the 'Submissions Needing Revision' folder to locate your manuscript file.

Please include the following items when submitting your revised manuscript:

- A rebuttal letter that responds to each point raised by the academic editor and reviewer(s). You should upload this letter as a separate file labeled 'Response to Reviewers'.
- A marked-up copy of your manuscript that highlights changes made to the original version. You should upload this as a separate file labeled 'Revised Manuscript with Track Changes'.
- An unmarked version of your revised paper without tracked changes. You should upload this as a separate file labeled 'Manuscript'.

If you would like to make changes to your financial disclosure, please include your updated statement in your cover letter. Guidelines for resubmitting your figure files are available below the reviewer comments at the end of this letter.

If applicable, we recommend that you deposit your laboratory protocols in protocols.io to enhance the reproducibility of your results. Protocols.io assigns your protocol its own identifier (DOI) so that it can be cited independently in the future. For instructions see: <http://journals.plos.org/plosone/s/submission-guidelines#loc-laboratory-protocols>

We look forward to receiving your revised manuscript.

Kind regards,

Alessio Paffoni, PhD
Academic Editor
PLOS ONE


Reviewers' comments:

Reviewer's Responses to Questions

**Comments to the Author**

1. If the authors have adequately addressed your comments raised in a previous round of review and you feel that this manuscript is now acceptable for publication, you may indicate that here to bypass the “Comments to the Author” section, enter your conflict of interest statement in the “Confidential to Editor” section, and submit your "Accept" recommendation.

Reviewer #1: All comments have been addressed

Reviewer #2: All comments have been addressed

**AUTHORS’ RESPONSE**

Thank you.

2. Is the manuscript technically sound, and do the data support the conclusions?

The manuscript must describe a technically sound piece of scientific research with data that supports the conclusions. Experiments must have been conducted rigorously, with appropriate controls, replication, and sample sizes. The conclusions must be drawn appropriately based on the data presented.

Reviewer #1: Yes

Reviewer #2: Yes

**AUTHORS’ RESPONSE**

Thank you.

3. Has the statistical analysis been performed appropriately and rigorously?

Reviewer #1: Yes

Reviewer #2: Yes

**AUTHORS’ RESPONSE**

Thank you.

4. Have the authors made all data underlying the findings in their manuscript fully available?

The [PLOS Data policy](http://www.plosone.org/static/policies.action#sharing) requires authors to make all data underlying the findings described in their manuscript fully available without restriction, with rare exception (please refer to the Data Availability Statement in the manuscript PDF file). The data should be provided as part of the manuscript or its supporting information, or deposited to a public repository. For example, in addition to summary statistics, the data points behind means, medians and variance measures should be available. If there are restrictions on publicly sharing data—e.g. participant privacy or use of data from a third party—those must be specified.

Reviewer #1: Yes

Reviewer #2: Yes

**AUTHORS’ RESPONSE**

Thank you.

5. Is the manuscript presented in an intelligible fashion and written in standard English?

PLOS ONE does not copyedit accepted manuscripts, so the language in submitted articles must be clear, correct, and unambiguous. Any typographical or grammatical errors should be corrected at revision, so please note any specific errors here.

Reviewer #1: Yes

Reviewer #2: Yes

**AUTHORS’ RESPONSE**

Thank you.

6. Review Comments to the Author

Please use the space provided to explain your answers to the questions above. You may also include additional comments for the author, including concerns about dual publication, research ethics, or publication ethics. (Please upload your review as an attachment if it exceeds 20,000 characters)

Reviewer #1: In Table 3, the reference should be stated for each of the independent variables. This will make it easier to interpret the odds ratios.

Reviewer #2: The authors adressed all the comments I did in the previous revision. They enlarged the information that was required. Many thanks.

**AUTHORS’ RESPONSE**

 As suggested, we have now indicated the reference value (Reference) as needed in table 3.

7. PLOS authors have the option to publish the peer review history of their article ([what does this mean?](https://journals.plos.org/plosone/s/editorial-and-peer-review-process#loc-peer-review-history)). If published, this will include your full peer review and any attached files.


If you choose “no”, your identity will remain anonymous but your review may still be made public.


**Do you want your identity to be public for this peer review?** For information about this choice, including consent withdrawal, please see our [Privacy Policy](https://www.plos.org/privacy-policy).

Reviewer #1: No

Reviewer #2: Yes: Lina María Garnica Rosas

**AUTHORS’ RESPONSE**

Thank you.

[NOTE: If reviewer comments were submitted as an attachment file, they will be attached to this email and accessible via the submission site. Please log into your account, locate the manuscript record, and check for the action link "View Attachments". If this link does not appear, there are no attachment files.]

While revising your submission, please upload your figure files to the Preflight Analysis and Conversion Engine (PACE) digital diagnostic tool, <https://pacev2.apexcovantage.com/>. PACE helps ensure that figures meet PLOS requirements. To use PACE, you must first register as a user. Registration is free. Then, login and navigate to the UPLOAD tab, where you will find detailed instructions on how to use the tool. If you encounter any issues or have any questions when using PACE, please email PLOS at [figures@plos.org](mailto:figures@plos.org). Please note that Supporting Information files do not need this step.
